# Machine learning-guided identification and simulation-based validation of potent JAK3 inhibitors for cancer therapy

**DOI:** 10.1371/journal.pone.0338777

**Published:** 2025-12-12

**Authors:** Hailang Wei, Qingyun Wang

**Affiliations:** Taihe Hospital, Affiliated Hospital of Hubei University of Medicine, Shiyan, China; Jamia Millia Islamia Central University: Jamia Millia Islamia, INDIA

## Abstract

Janus kinase 3 (JAK3) is a hematopoietic-specific kinase implicated in cytokine signaling and immune dysregulation and has recently been associated with cancer progression. However, selective and potent JAK3 inhibitors remain underdeveloped. In this study, we established a machine learning (ML)-based pipeline to identify novel JAK3 inhibitors with anti-cancer potential. A curated ChEMBL dataset of JAK3 inhibitors was used to train multiple ML classifiers, with the Random Forest model achieving the highest performance (AUC = 0.80, F1-score = 0.92). This model was applied to virtually screen 25,084 ChEMBL compounds, yielding 400 high-confidence candidates (prediction score > 0.9). Docking analysis identified ten top binders (binding affinity ≤ –8.5 kcal/mol), of which three CHEMBL49087, CHEMBL4117527, and CHEMBL50064 exhibited optimal ADMET profiles. These compounds underwent 200 ns molecular dynamics simulations, showing low RMSD (0.10–0.20 nm), stable binding conformations, and preserved protein compactness. MM/GBSA calculations revealed that CHEMBL4117527 displayed the strongest binding free energy (–29.5 kcal/mol), surpassing even the co-crystallized ligand (–17.7 kcal/mol). Our integrative approach combining machine learning, docking, pharmacokinetics, molecular dynamics, and free energy analysis presents a robust computational strategy for JAK3 inhibitor discovery. These findings support CHEMBL4117527 as promising candidates for further experimental evaluation in cancer therapeutics.

## 1. Introduction

Janus kinase 3 (JAK3) is a non-receptor tyrosine kinase belonging to the Janus kinase (JAK) family, which also includes JAK1, JAK2, and TYK2. Among these, JAK3 is unique due to its exclusive expression in hematopoietic cells and its specific interaction with the common gamma chain (γc) shared by cytokine receptors such as interleukin (IL)-2, IL-4, IL-7, IL-9, IL-15, and IL-21 [1,2]. Through this signalling axis, JAK3 plays a central role in lymphocyte proliferation, differentiation, and immune homeostasis [3]. Dysregulation of JAK3 signalling, either through gain-of-function mutations or constitutive activation, has been implicated in a variety of immune disorders and malignancies, including acute lymphoblastic leukemia, T-cell prolymphocytic leukemia, cutaneous T-cell lymphoma, and certain solid tumors [4–6]. In contrast to other JAK family members that are ubiquitously expressed, JAK3’s restricted distribution to hematopoietic and immune cells offers a therapeutic advantage—selective inhibition of JAK3 can modulate pathological immune responses or malignant signalling without widespread systemic immunosuppression [7]. However, designing selective JAK3 inhibitors remains challenging due to the high homology of ATP-binding sites among JAK kinases, particularly between JAK1 and JAK2 [8]. This underscores the need for rational design strategies that prioritize selectivity, safety, and minimized off-target effects, which have limited the clinical utility of first-generation pan-JAK inhibitors such as tofacitinib and ruxolitinib [9,10]. Recent studies have further revealed the involvement of JAK3 in tumors immune evasion and microenvironmental crosstalk, suggesting that JAK3-targeted therapy could not only suppress oncogenic signalling but also restore anti-tumor immunity [11,12]. Therefore, selective inhibition of JAK3 represents a promising yet underexplored approach in cancer therapeutics, with potential applications extending beyond hematologic malignancies to solid tumors exhibiting aberrant JAK/STAT pathway activation [13–15]. Traditional drug discovery approaches for kinase inhibitors, although effective, are resource-intensive and time-consuming. The integration of machine learning (ML) and computer-aided drug design (CADD) now enables high-throughput identification of bioactive compounds by learning molecular patterns that correlate with inhibitory potency [16,17]. ML-based strategies have demonstrated particular success in virtual screening, structure–activity relationship (SAR) modelling, and activity prediction for kinase families, yet most studies stop at prediction and lack structural and energetic validation [18,19]. To overcome these limitations, structure-based modelling and physics-driven simulations provide complementary insights. Molecular docking elucidates binding orientations and interaction energetics, while molecular dynamics (MD) simulations capture the conformational stability and flexibility of protein–ligand complexes under physiological conditions [20]. Furthermore, quantum chemical methods, particularly density functional theory (DFT), allow for precise evaluation of frontier molecular orbitals (HOMO/LUMO) and global reactivity descriptors, aiding in the optimization of electronic and physicochemical properties of potential inhibitors [21]. Despite increasing interest in AI-driven kinase inhibitor discovery, few studies have integrated ML, docking, MD simulations, and DFT into a single, end-to-end workflow focused on JAK3 selectivity and stability. Previous efforts have largely been limited to predictive modelling or docking-based screening, without multi-level validation. To bridge this gap, the present study establishes a machine learning-guided and simulation-validated computational pipeline for identifying potent and selective JAK3 inhibitors. Specifically, we (i) curated and modelled a dataset of JAK3 inhibitors using advanced ML algorithms, (ii) performed virtual screening and molecular docking to assess binding interactions, (iii) evaluated dynamic stability through MD simulations and free energy analysis, and (iv) characterized quantum chemical descriptors via DFT to elucidate electronic reactivity and stability.

## 2. Methodology

### 2.1. Dataset acquisition and preprocessing

To construct predictive models for Janus kinase 3 (JAK3) inhibitors, compound data were extracted from the ChEMBL database [22]. The initial dataset was refined to retain only entries corresponding to binding assays (assay type ‘B’), ensuring that only compounds with direct interaction evidence with the target protein were considered. Data records with undefined or missing IC₅₀ values, or where the activity relation was not equal to ‘=’, were removed to maintain consistency in activity thresholds. Duplicate entries, identified based on canonical SMILES, were also excluded to avoid bias from repeated structures. A binary classification scheme was applied: compounds with IC₅₀ values ≤ 500 nM were labeled as inhibitors (active, class 0), while those with IC₅₀ values between 501 and 10,000 nM were labeled as non-inhibitors (inactive, class 1). Compounds with IC₅₀ values exceeding 10,000 nM were discarded as outliers. The cleaned dataset was randomly divided into an 80:20 ratio for training and internal validation, maintaining label proportions through stratified sampling. The withheld 20% served as an external validation set to evaluate model generalizability. Principal component analysis (PCA) was conducted on normalized molecular weight data to examine the chemical diversity and potential clustering of active and inactive compounds within the feature space.

### 2.2. Descriptor generation and feature refinement

Molecular descriptors were generated using MACCS (Molecular ACCess System) structural keys, which encode 166 binary features representing the presence or absence of predefined substructures [23]. These fingerprints are well-established in cheminformatics due to their interpretability and computational efficiency. To reduce dimensionality and enhance model interpretability, Recursive Feature Elimination (RFE) was applied using a Random Forest classifier as the estimator [24]. RFE iteratively eliminates less informative features, retaining the subset that contributes most significantly to classification performance. This step helps minimize the risks of overfitting and improves training efficiency.

### 2.3. model development and evaluation strategy

The scikit-learn library in python was used to train four supervised learning models including Random Forest (RF), Decision Tree (DT), Naïve Bayes (NB), and Support Vector Machine (SVM). Each model utilized the selected MACCS descriptors as input features.

- **Decision Tree** builds hierarchical decision rules based on feature thresholds to separate the classes.
- **Random Forest** constructs decision trees ensemble and aggregates ensemble predictions, reducing overfitting and variance.
- **Support Vector Machine** maps data into higher-dimensional space to find an optimal separating hyperplane, making it suitable for non-linearly separable data [25].
- **Naïve Bayes** applies probabilistic learning under the assumption of conditional independence among features.

Performance was assessed using several metrics: accuracy, precision, recall, F1-score, and area under the ROC curve (AUC). These metrics were computed on both the internal validation split and the external test set to evaluate model robustness and generalization. The formulas used are:


$$Precision=\frac{\text{TP}}{TP+FP}$$

$$Accuracy=\frac{\text{TP}+\text{TN}}{\text{TP}+\text{TN}+\text{FP}+\text{FN}}$$

$$Recall=\frac{\text{TP}}{\text{TP}+\text{FN}}$$

$$F\text{1}-score=\frac{\text{2}\;\times \text{Precision}\;\times \text{Recall}}{\text{Precision}+\text{Recall}}$$

Where TP, TN, FP, and FN denote true positives, true negatives, false positives, and false negatives respectively.

### 2.4. Molecular docking

To evaluate the binding potential of the top-ranked hits screened through machine learning, molecular docking studies were conducted using the Glide module of Schrödinger Maestro Suite [26]. The crystal structure of human JAK3 protein (PDB ID: 3LXL) was retrieved from the Protein Data Bank. Missing residues were modeled using AlphaFold2-based structure prediction to complete the target structure [27]. Protein preparation was performed using the Protein Preparation Wizard in Maestro, which included assigning bond orders, adding missing hydrogen atoms, optimizing hydrogen bonding networks, and minimizing the structure using the OPLS force field [28]. Ligand molecules were energy minimized and converted to 3D conformations, followed by preparation using LigPrep. Docking was carried out using Glide in standard precision (SP) mode [29]. The receptor grid was generated at the centroid of the co-crystallized ligand binding site, and default settings were used with flexible ligand sampling. The top binding poses were selected based on Glide docking scores and visual inspection of interaction profiles.

### 2.5. ADMET evaluation

To assess the pharmacokinetic and safety profiles of the selected JAK3 inhibitors, in silico ADMET (Absorption, Distribution, Metabolism, Excretion, and Toxicity) analysis was performed. Key physicochemical properties including Lipinski’s rule of five, gastrointestinal absorption, blood-brain barrier permeability was predicted using the SwissADME online tool [30]. Toxicity risk assessments such as mutagenicity and tumorigenicity were evaluated using OSIRIS Property Explorer [31]. Compounds with favorable ADMET profiles were prioritized for further molecular dynamics studies.

### 2.6. Molecular dynamics simulations

To investigate the stability and conformational dynamics of ligand–JAK3 complexes, molecular dynamics (MD) simulations were performed with GROMACS 2023.3 [32]. System preparation was carried out through the CHARMM-GUI interface, which generated the protein and ligand topologies based on the CHARMM36 all-atom force field [33]. Each complex was placed in a cubic periodic box and solvated with TIP3P water molecules, followed by neutralization with appropriate numbers of K⁺ and Cl⁻ ions. Energy minimization was conducted using the steepest descent algorithm to relax unfavorable contacts. The equilibrations were carried out in two stages: a 100 ps NVT run to stabilize temperature and a 100 ps NPT run to equilibrate pressure, both maintained at 303 K and 1 bar. Subsequently, a 200 ns production simulation was executed with a 2-fs integration step. Trajectory analyses included the calculation of root mean square deviation (RMSD); root mean square fluctuation (RMSF), radius of gyration (Rg), and solvent accessible surface area (SASA) to assess structural stability and flexibility. In addition, MM/GB(PB)SA free energy calculations were carried out using the gmx_MMPBSA package on snapshots extracted from the final 10 ns of each trajectory to evaluate binding energetics [34].

### 2.7. Density functional theory (DFT) calculations

The selected compounds were subjected to quantum chemical calculations to analyse their frontier molecular orbitals (HOMO and LUMO) and global reactivity descriptors. Geometry optimization of each compound was carried out using the Density Functional Theory (DFT) method with the B3LYP functional and the 6-311G(d,p) basis set, as implemented in the Gaussian 16 software package [35]. The calculations were performed in an aqueous environment using the self-consistent reaction field (SCRF) approach to simulate solvent effects, thereby providing a realistic representation of the compounds’ behaviour under physiological conditions. Default convergence criteria were applied, and no symmetry constraints were imposed during optimization.

### 2.8. Toxicity prediction analysis

The toxicity profiles of the selected compounds were evaluated using the ProTox-II web server (https://tox-new.charite.de/protox_II/), an established platform for *in silico* prediction of various toxicity endpoints based on machine learning–derived models. The SMILES structures of the compounds were uploaded to the server, and multiple toxicity parameters were predicted, including LD₅₀ (median lethal dose), cytotoxicity, carcinogenicity, and immunotoxicity.

## 3. Results

### 3.1. Distribution of molecular weights and PCA

**Fig 1:**
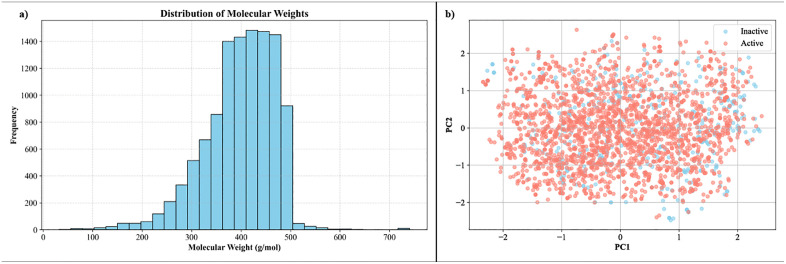
Distribution of Molecular Weights and PCA Analysis of modelling data. (a) Molecular weight distribution, with a peak around 400 g/mol. (b) PCA plot showing clustering of active (red) and inactive (blue) compounds.

The distribution of molecular weights for the compounds analyzed is shown in Fig 1a. A prominent peak is observed around 400 g/mol, suggesting that a significant number of the compounds fall within the optimal molecular weight range for drug-like properties. This range is important for oral bioavailability and absorption, as it balances sufficient size for molecular interaction while maintaining favorable permeability across biological membranes. To further explore the chemical diversity of the dataset, Fig 1b presents a principal component analysis (PCA) plot, which visualizes the separation of active and inactive compounds. In this analysis, compounds classified as active (red) and inactive (blue) are plotted along the first two principal components, capturing the most significant variations in the dataset. While some overlap is evident between the two classes, a general clustering trend is observed, indicating that molecular characteristics contribute to distinguishing active from inactive compounds. This clustering provides valuable insight into the structural diversity and potential features that differentiate the active inhibitors from non-inhibitors.

### 3.2. Model performance evaluation

**Fig 2:**
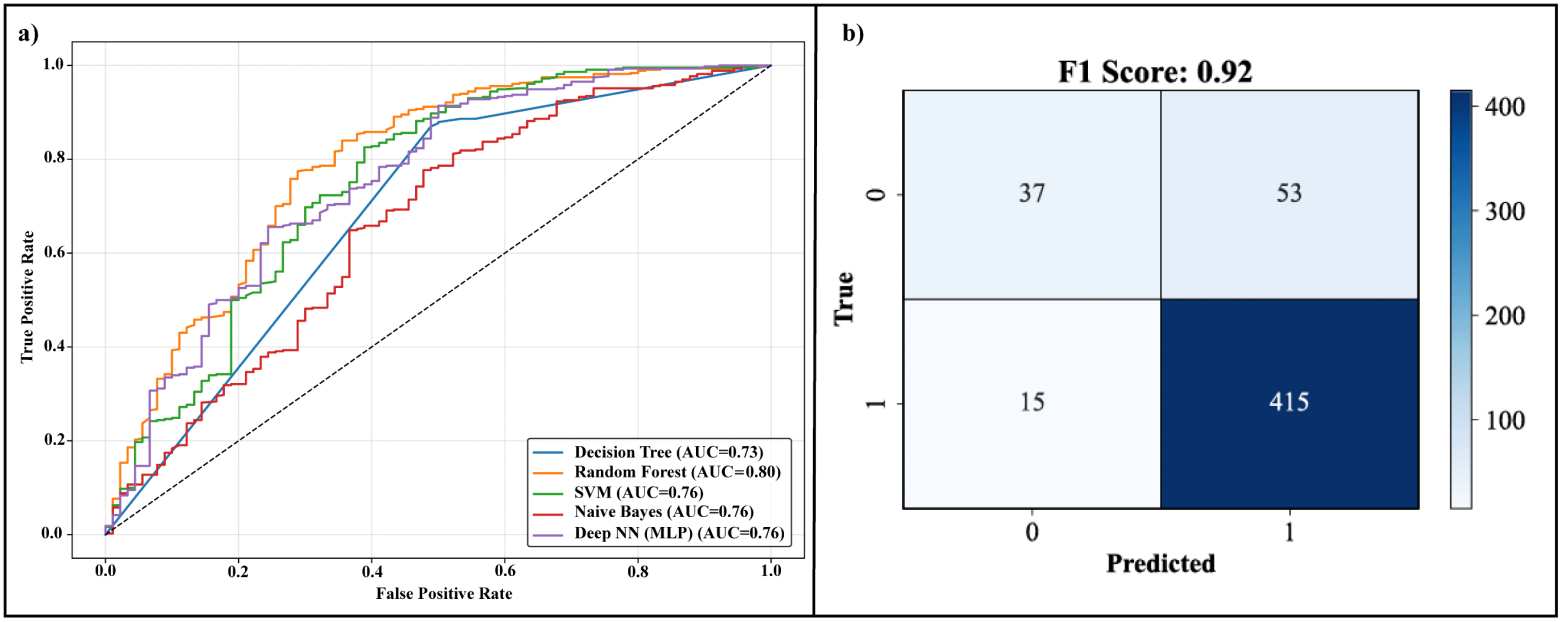
Model Performance Evaluation. (a) ROC curves for Decision Tree, Random Forest, SVM, Naïve Bayes, and Deep NN models. (b) Confusion matrix for Random Forest, with an F1 score of 0.92.

To evaluate the ability of machine learning models to classify JAK3 inhibitors, four algorithms Decision Tree, Random Forest, Support Vector Machine (SVM), and Naïve Bayes were employed. The models were assessed using several performance metrics: accuracy, precision, recall, and F1-score (Table 1). Fig 2a illustrates the ROC curves for each model. Among the models tested, Random Forest exhibited the highest performance, achieving an area under the curve (AUC) of 0.80. This indicates that Random Forest has a high ability to discriminate between active and inactive compounds, showing the best trade-off between sensitivity and specificity. The Support Vector Machine (SVM) and Deep NN follow closely with an AUC of 0.76, demonstrating strong classification ability but slightly lower performance than Random Forest. Decision Tree and Naïve Bayes showed lower discriminative power, with AUC values of 0.73 and 0.65, respectively, reflecting their limited effectiveness in handling the complexity of the dataset. The confusion matrix for the Random Forest model is shown in Fig 2b. With an F1 score of 0.92, the model demonstrated excellent performance in balancing precision and recall, ensuring that both false positives and false negatives were minimized. Precision (0.87) indicates that a significant proportion of the predicted active compounds were indeed active, while the recall (0.96) highlights the ability of model to identify most of the true active compounds. This suggests that Random Forest is highly reliable in identifying JAK3 inhibitors while avoiding misclassifications.

**Table 1:** Comparative assessment of four machine learning models using stratified 10-fold cross-validation.

Model	TP	FP	TN	FN	Accuracy	Precision	Recall	F1-score
Decision Tree	378	45	45	52	0.81	0.89	0.87	0.88
Random Forest	415	59	31	15	0.85	0.87	0.96	0.92
SVM	426	68	22	4	0.86	0.86	0.99	0.92
Naïve Bayes	352	49	41	78	0.75	0.87	0.81	0.84
Deep NN	426	68	22	4	0.86	0.86	0.99	0.92

### 3.3. External validation of random forest model

**Fig 3:**
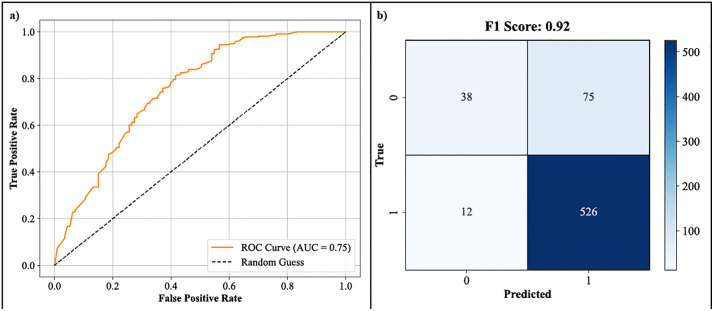
External Validation of Random Forest Model. ROC curve for external validation of Random Forest, showing an AUC of 0.75 and an F1 score of 0.92.

To assess the robustness and generalizability of the Random Forest model, an external validation was performed on a separate test set. The external validation results are presented in Fig 3, which shows the ROC curve for the external dataset. The model performed well, maintaining a high AUC of 0.75, indicating that the model’s ability to distinguish between active and inactive compounds is consistent across different datasets. This result suggests that the model is not overfitting to the training data and can generalize well to unseen compounds. Further analysis of the confusion matrix for the external validation data shows that the F1 score remains 0.92, like the internal validation results. This consistent performance across both training and external datasets confirms the robustness of the Random Forest model and its potential for real-world applications. The model’s ability to maintain a high level of accuracy on unseen data is critical for its applicability in the drug discovery process, where the validation set represents, compounds do not present in the training data.

### 3.4. Virtual screening of the ChEMBL database

To identify potential JAK3 inhibitors, the best-performing machine learning model Random Forest, was employed to virtually screen a library of 25,084 compounds retrieved from the ChEMBL database. Given that fingerprint-based machine learning models capture structural features that influence bioactivity, compounds predicted as active are expected to share relevant substructural similarities with those in the training dataset. Out of the screened compounds, a total of 400 molecules received prediction scores exceeding 0.9, indicating a high likelihood of JAK3 inhibitory activity. These high-confidence candidates were subsequently selected for structure-based validation through molecular docking studies.

### 3.6. Molecular docking analysis

**Fig 4:**
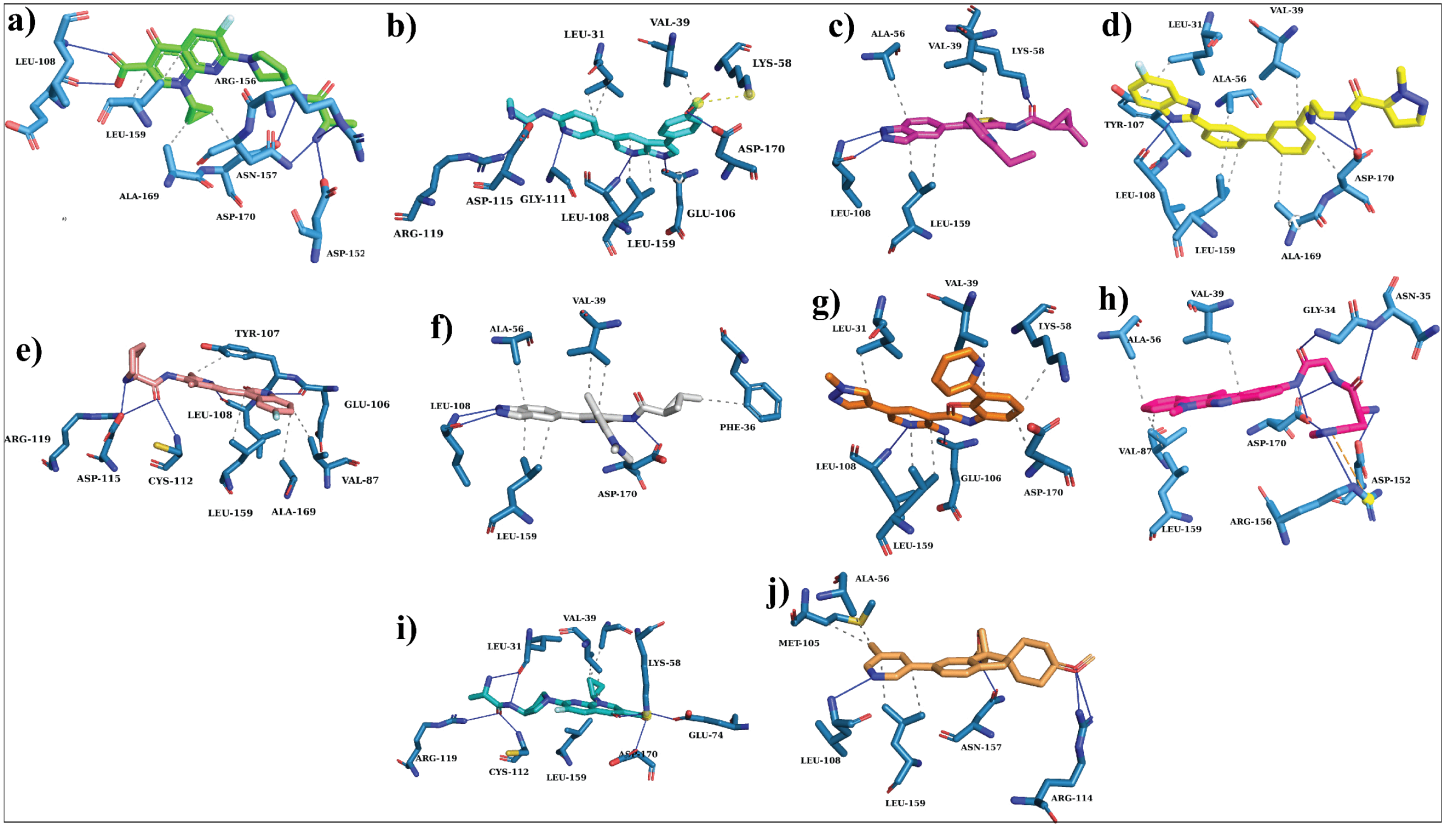
Visualization of key protein-ligand interactions, highlighting hydrogen bonds and hydrophobic contacts for the ten best-docked compounds. (a) CHEMBL49087, (b) CHEMBL537096, (c) CHEMBL2365364, (d) CHEMBL533775, (e) CHEMBL4117527, (f) CHEMBL2364957, (g) CHEMBL2078655, (h) CHEMBL2220288, (i) CHEMBL50064, (j) CHEMBL4116294.

To validate the virtual screening results and assess the binding potential of high-scoring compounds, molecular docking was carried out using the Glide module in the Schrödinger Maestro Suite. The top 400 predicted JAK3 inhibitors from the ChEMBL database were docked against the JAK3 protein structure. Docking scores were used to rank the compounds, and the ten best binders were selected for further interaction analysis (Table 2). CHEMBL49087 exhibited the strongest binding affinity of –9.903 kcal/mol, forming six hydrogen bonds with Glu106, Leu108, Arg156, Asn157, Asp170, and Asp152, with bond lengths ranging between 1.73 and 2.69 Å. Key hydrophobic contacts were observed with Ala56, Leu156, and Met105, suggesting deep burial into the JAK3 binding pocket. CHEMBL537096 followed closely with a binding energy of –9.824 kcal/mol, interacting via hydrogen bonds with Asp170, Glu106, Leu108, Arg119, Asp115, and Lys58. The ligand formed additional hydrophobic contacts with Ala56, Leu159, Val39, and Gly111. Other strong candidates included CHEMBL2365364, CHEMBL533775, and CHEMBL4117527, with binding affinities of –9.168, –9.13, and –9.051 kcal/mol, respectively. These compounds exhibited several hydrogen bonding interactions involving residues such as Lys58, Leu108, Cys112, and Asp115, with additional van der Waals contacts stabilizing the complexes. Notably, compounds like CHEMBL2364957 and CHEMBL2078655 also showed favorable binding energies (–8.986 and –8.886 kcal/mol) and interacted with important residues such as Cys112, Leu159, and Met105, which are implicated in JAK3 ligand recognition. Overall, the molecular docking results (Fig 4) reveal that the selected compounds establish strong interactions within the JAK3 active site, supported by multiple hydrogen bonds and hydrophobic contacts. These findings suggest favorable binding orientations and support the selection of these molecules for subsequent molecular dynamics simulations.

**Table 2:** Binding affinities and interaction profiles of top 10 docked JAK3 inhibitors.

Compounds	Binding Affinity (kcal/mol)	Hydrogen Bonds (Bond length)	Other Interactions
CHEMBL49087	−9.903	Glu106(1.76), Leu108(2.07), Arg156(2.26), Asn157(2.69), Asp170(1.83), Asp152(1.73)	Ala56, Leu156, Met105, Val39, Ala169, Leu31
CHEMBL537096	−9.824	Asp170(1.74), Ly58(2.20), Glu106(2.20), Leu108(2.13), Arg119(2.76), Asp115(2.32)	Ala169, Val39, Val87, Met105, Ala56, Leu159, Leu31, Gly111, Tyr106
CHEMBL2365364	−9.168	Lys58(2.34), Leu108(1.70)	Met105, Tyr107, Leu159, Ala56, Ala169, Val39
CHEMBL533775	−9.13	Leu108(2.05), Asp170(1.82)	Asn35, Asp152, Asn157, Met105, Ala169, Val39, Ala56, Leu159, Gly111
CHEMBL4117527	−9.051	Glu106(2.38), Leu108(2.12), Cys112(2.48), Asp115(1.85)	Ala169, Leu159, Val39, Val87, Ala56, Leu31, Tyr107, Met105, Gly111
CHEMBL2364957	−8.986	Leu108(2.03)	Phe36, Asn157, Arg156, Cys112, Val39, Tyr107, Leu159, Ala56, Lys58, Met105, Ala169
CHEMBL2078655	−8.886	Glu106(1.97), Leu108(2.25)	Val39, Lys58, Met105, Ala169, Leu159, Ala565, Tyr107, Leu31, Gly111
CHEMBL2220288	−8.854	Asp152(1.89), Asn157(2.46), Asp170(2.13)	Leu31, Val39, Ala56, Leu159, Met105, Val87, Gly34, Ala169, Arg156
CHEMBL50064	−8.581	Leu31(2.41), Lys58(1.96), Glu74(2.08), Cy112(2.10), Asp170(2.01)	Gly111, Ala56, Leu159, Met105, Ala169, Val39
CHEMBL4116294	−8.51	Leu108(2.16), Arg114(1.85), Asn157(2.13)	Leu31, Leu159, Glu106, Ala56, Met105, Val87, Cys112

### 3.7. ADMET evaluation

**Fig 5:**
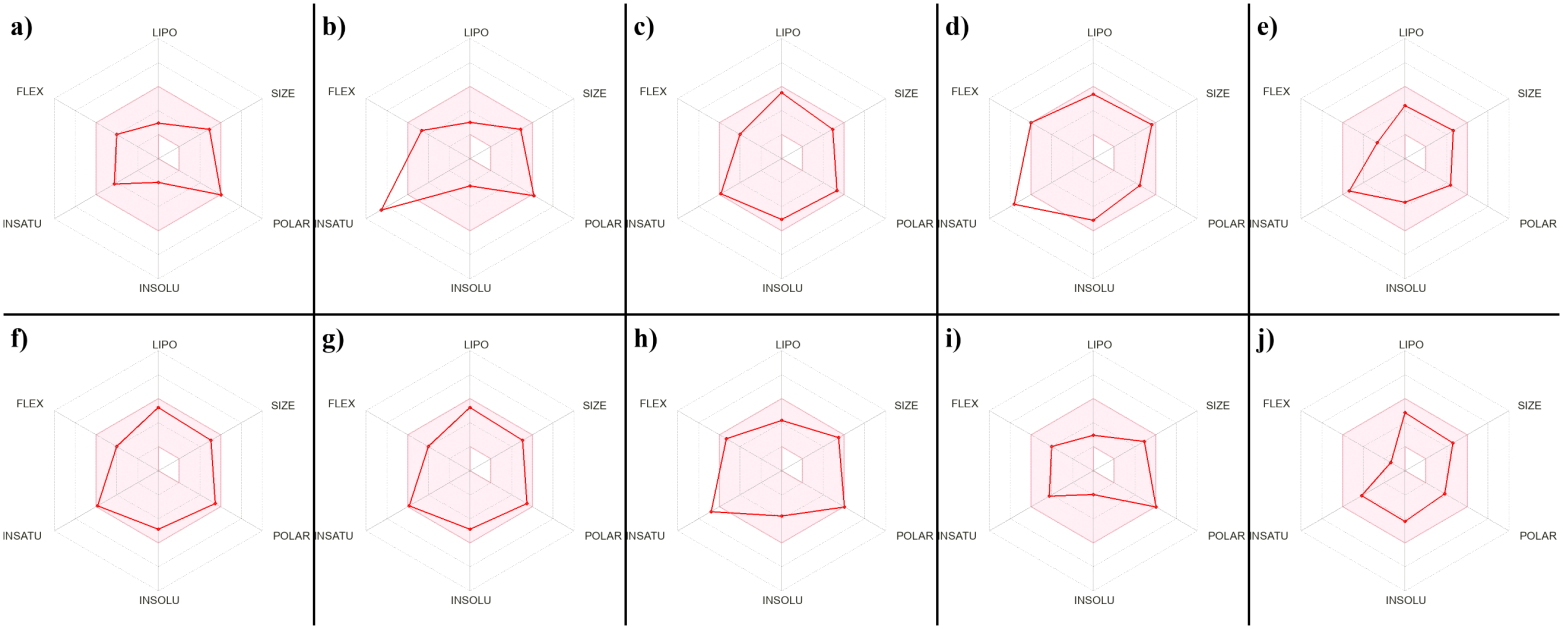
Radar plots of physicochemical descriptors for top 10 docked compounds. (a) CHEMBL49087, (b) CHEMBL537096, (c) CHEMBL2365364, (d) CHEMBL533775, (e) CHEMBL4117527, (f) CHEMBL2364957, (g) CHEMBL2078655, (h) CHEMBL2220288, (i) CHEMBL50064, (j) CHEMBL4116294.

Following molecular docking, the top-performing compounds were subjected to ADMET analysis to assess their drug-likeness and pharmacokinetic suitability. A total of ten compounds were evaluated using SwissADME and OSIRIS Property Explorer tools, and the results are presented in Tables 3 and 4. All compounds showed high gastrointestinal absorption (GIA) and satisfied Lipinski’s rule of five, Veber, and Egan filters (Table 3). Most also passed the Ghose filter, except for CHEMBL2220288, which failed due to deviations in molar refractivity. Drug-likeness radar plots for each compound (Fig 5) illustrate the balance across six key descriptors lipophilicity (LIPO), size, polarity (POLAR), solubility (INSOLU), saturation (INSATU), and flexibility (FLEX). In terms of toxicity profiles, most compounds showed no risk of mutagenicity, tumorigenicity, irritation, or reproductive toxicity. However, CHEMBL2365364 and CHEMBL2364957 raised concerns regarding potential mutagenicity, and CHEMBL2220288 showed a high risk for reproductive toxicity (Table 4). Based on the overall safety and ADMET profiles, CHEMBL49087, CHEMBL4117527, and CHEMBL50064 were selected for further molecular dynamics (MD) simulations. The Drug-Score metric—which integrates drug-likeness, toxicity, and pharmacokinetic properties—highlighted CHEMBL49087 and CHEMBL50064 (score = 0.73), and CHEMBL4117527 (score = 0.70) as the most promising candidates.

**Table 3:** Physicochemical and drug-likeness properties of top JAK3 inhibitors.

Compounds	GIA	Lipinski	Ghose	Veber	Egan	Drug Score
CHEMBL49087	High	Yes	Yes	Yes	Yes	0.73
CHEMBL537096	High	Yes	Yes	Yes	No	0.28
CHEMBL2365364	High	Yes	Yes	Yes	Yes	0.38
CHEMBL533775	High	Yes	Yes	Yes	No	0.45
CHEMBL4117527	High	Yes	Yes	Yes	Yes	0.7
CHEMBL2364957	High	Yes	Yes	Yes	Yes	0.31
CHEMBL2078655	High	Yes	Yes	Yes	Yes	0.53
CHEMBL2220288	High	Yes	No	Yes	Yes	0.39
CHEMBL50064	High	Yes	Yes	Yes	Yes	0.73
CHEMBL4116294	High	Yes	Yes	Yes	Yes	0.38

**Table 4:** Predicted toxicity profiles of top JAK3 inhibitor candidates.

Compounds	Mutagenic	Tumorigenic	Irritant	Reproductive Effect
CHEMBL49087	No	No	No	No
CHEMBL537096	No	No	No	No
CHEMBL2365364	High	No	No	No
CHEMBL533775	No	No	No	No
CHEMBL4117527	No	No	No	No
CHEMBL2364957	High	No	No	Mild
CHEMBL2078655	No	No	No	No
CHEMBL2220288	No	No	No	High
CHEMBL50064	No	No	No	No
CHEMBL4116294	No	No	No	No

### 3.8. Binding stability analysis

**Fig 6:**
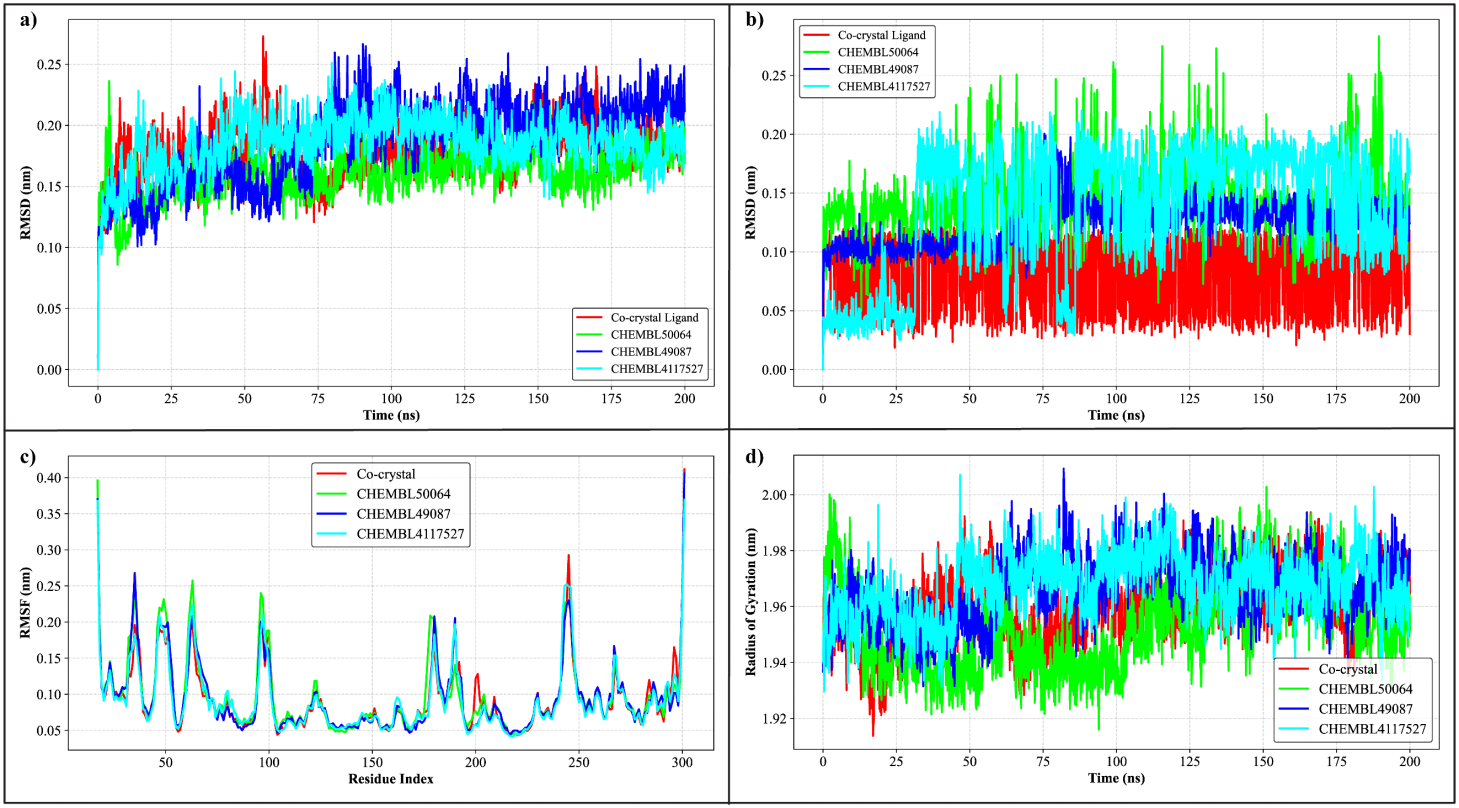
Molecular dynamics analysis of JAK3–ligand complexes over a 200 ns simulation. (a) RMSD of JAK3 complexes. (b) RMSD of ligand atoms. (c) RMSF analysis. (d) Radius of gyration profiles of complexes.

**Fig 7:**
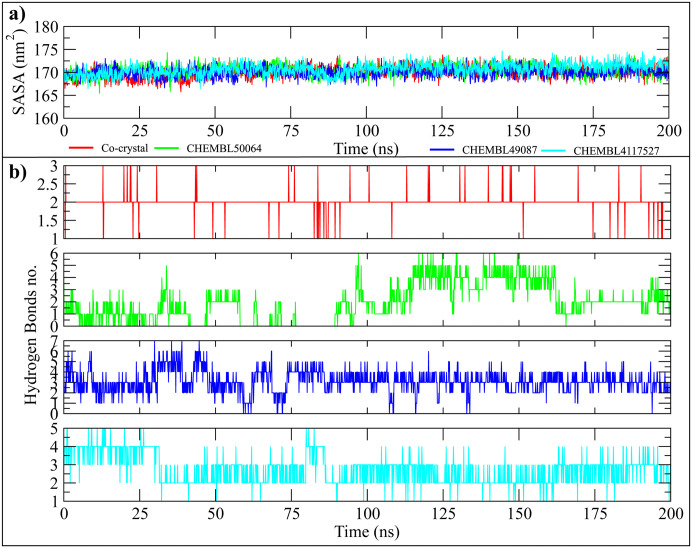
SASA and Hydrogen Bond Analysis for JAK3-Ligand Complexes over 200 ns Simulations. (a) SASA profiles of the co-crystal and top ligands (CHEMBL50064, CHEMBL49087, and CHEMBL4117527). (b) Hydrogen bond formation between the protein and the ligands throughout the simulation.

To evaluate the dynamic behavior and structural stability of the protein–ligand complexes, molecular dynamics (MD) simulations were extended to 200 ns using the GROMACS 2023.3 simulation package for CHEMBL49087, CHEMBL4117527, CHEMBL50064, and the co-crystallized JAK3-ligand complex. Several trajectory analyses were conducted to assess the temporal evolution of each system. The RMSD of the carbon alpha atoms was first computed to evaluate the global stability of the JAK3 structure. As shown in Fig 6a, all systems achieved equilibrium within the first 10–20 ns of the simulation and fluctuated minimally thereafter. The RMSD values remained between 0.10 and 0.20 nm for most of the trajectory. Among the candidates, CHEMBL50064 exhibited the lowest RMSD (~0.12 nm average), closely followed by CHEMBL49087 (~0.13 nm) and CHEMBL4117527 (~0.14 nm), which were comparable to the co-crystal (~0.13 nm), suggesting well-maintained structural integrity throughout the 200 ns simulation. To monitor ligand mobility within the binding site, ligand RMSD values were computed relative to their initial docked positions (Fig 6b). All ligands remained tightly anchored, with CHEMBL49087 and CHEMBL4117527 showing the most stable behavior, maintaining RMSD values between 0.08 and 0.15 nm. CHEMBL50064 exhibited slightly higher fluctuations (~0.17 nm at its peak), but remained within acceptable bounds, confirming minimal displacement and persistent binding within the active site. Moreover, the snapshots of md trajectory were retrieved after every 20 ns and aligned to find the ligand stability within the binding site. The analysis revealed that all ligands remained stably bound with the active site (Supplementary Figure 1 in S1 Data). The RMSF per residue was analyzed to assess local flexibility within the protein (Fig 6c). All systems showed similar fluctuation trends, with elevated RMSF values observed at the N-terminal (residues 1–20), loop regions (~residues 130–150), and C-terminal (~residues 290–300), as expected for solvent-exposed or disordered regions. In contrast, core binding site residues (e.g., Leu108, Arg156, Asn157, and Asp170) remained highly stable, with RMSF values consistently below 0.15 nm, indicating preserved binding interactions. To quantify the overall compactness of the protein during simulation, the radius of gyration (Rg) was calculated (Fig 6d). All complexes maintained steady Rg values within the range of 1.94–1.98 nm. CHEMBL50064 exhibited the most compact structure (avg. Rg ≈ 1.95 nm), slightly more stable than CHEMBL4117527 and CHEMBL49087 (both averaging ~1.96 nm), and comparable to the co-crystallized reference. Additionally, SASA was evaluated to examine the extent of protein exposure to solvent molecules over time (Fig 7a). All systems showed consistent SASA values between 168 and 172 nm², with minimal fluctuations, suggesting stable protein folding and solvent interaction dynamics. Specifically, CHEMBL4117527 had the highest average SASA (171.2 nm²), followed by CHEMBL49087 (170.5 nm²) and CHEMBL50064 (169.7 nm²), which were all comparable to the co-crystal (169.9 nm²). The number of hydrogen bonds formed between the ligands and the protein was monitored throughout the simulation. As shown in Fig 7b, CHEMBL49087 and CHEMBL4117527 maintained a stable number of hydrogen bonds (ranging between 3–5 bonds), with occasional transient breaks, especially in the first 50 ns. CHEMBL50064 exhibited a slightly lower and more variable number of hydrogen bonds (ranging between 2–4 bonds), with more frequent disruptions compared to the other compounds, which may contribute to its higher RMSD fluctuations. Furthermore, the protein-ligand contacts were compared from start and end frames, showing conserved residues involved in interactions with hit compounds (Supplementary Figure 2 in S1 Data). These results suggest that CHEMBL49087 and CHEMBL4117527 establish more stable interactions with the protein than CHEMBL50064.

### 3.9. MM/GBSA binding free energy analysis

**Fig 8:**
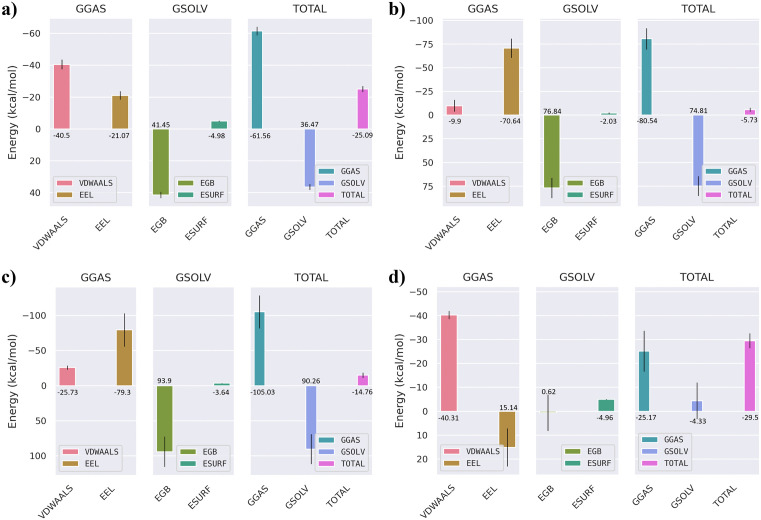
MM/GBSA binding free energy of JAK3–ligand complexes. Total and component energies (kcal/mol) for: (a) Co-crystallized ligand, (b) CHEMBL50064, (c) CHEMBL49087, and (d) CHEMBL4117527.

**Fig 9:**
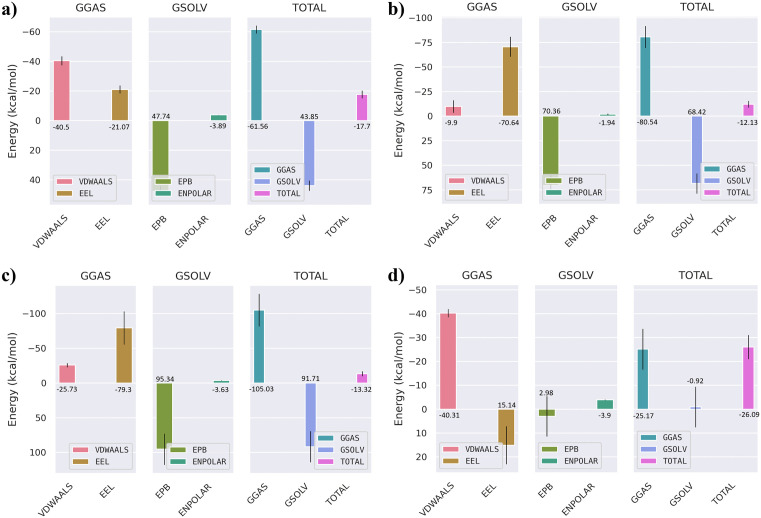
MM/PBSA binding free energy of JAK3–ligand complexes. Total and component energies (kcal/mol) for: (a) Co-crystallized ligand, (b) CHEMBL50064, (c) CHEMBL49087, and (d) CHEMBL4117527.

**Fig 10:**
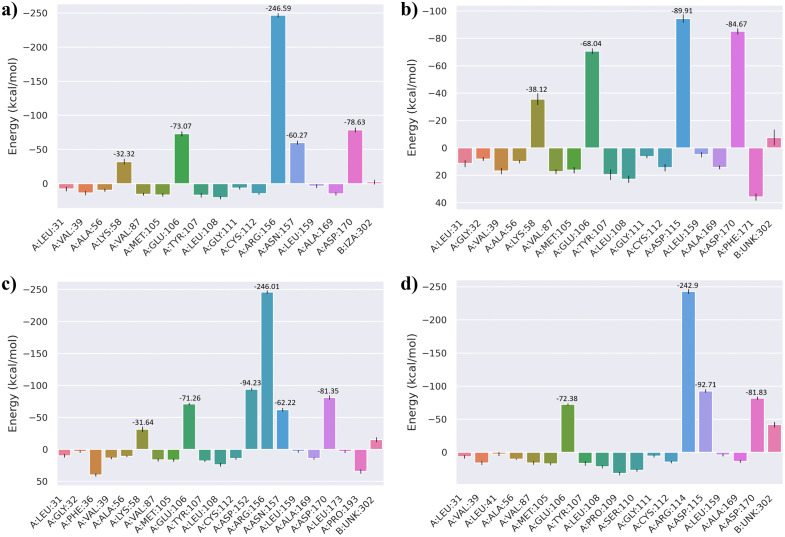
Per-residue free energy decomposition of JAK3–ligand complexes. (a) Co-crystallized ligand, (b) CHEMBL50064, (c) CHEMBL49087, and (d) CHEMBL4117527.

To further quantify the binding affinities and energetic stability of the selected compounds with the JAK3 protein, both MM/GBSA (Molecular Mechanics/Generalized Born Surface Area) and MM/PBSA (Molecular Mechanics/Poisson–Boltzmann Surface Area) calculations were performed using the last 10 ns of the 200 ns molecular dynamics trajectories. This timeframe was selected to ensure structural convergence and representative sampling of equilibrium states. The overall free energy components, including van der Waals, electrostatic, polar solvation, and non-polar solvation contributions, were evaluated to understand the driving forces governing ligand binding. As shown in Fig 8, the MM/GBSA results indicated that the co-crystallized ligand exhibited a total binding free energy of approximately –25.09 kcal/mol, dominated by van der Waals (–40.5 kcal/mol) and electrostatic (–21.07 kcal/mol) interactions, partially offset by the polar solvation penalty (41.45 kcal/mol). Among the screened hits, CHEMBL4117527 demonstrated the most favorable ΔG_bind_ (–29.5 kcal/mol), followed by CHEMBL49087 (–5.73 kcal/mol) and CHEMBL50064 (–25.09 kcal/mol). The stronger affinity of CHEMBL4117527 can be attributed to a balanced contribution from van der Waals (–40.31 kcal/mol) and electrostatic (15.14 kcal/mol) components, along with minimal desolvation penalties. This energetic pattern supports the enhanced stability observed during the MD trajectory. To validate the MM/GBSA results, complementary MM/PBSA calculations were performed (Fig 9). Both methods yielded consistent energetic profiles, with CHEMBL4117527 again showing the most stable complex (ΔG_bind _= –26.09 kcal/mol), driven by favorable van der Waals (–40.31 kcal/mol) and electrostatic (15.14 kcal/mol) interactions. The agreement between GB and PB models underscores the robustness of the binding free energy estimates and indicates that hydrophobic interactions play a major role in stabilizing the JAK3–ligand complexes. Per-residue energy decomposition (Fig 10) was carried out to identify amino acids contributing significantly to ligand stabilization within the binding pocket. Key residues such as Leu-31, Val-39, Met-87, Glu-106, Arg-155, and Leu-159 showed highly favorable energy contributions across all complexes, highlighting their central role in mediating hydrophobic and electrostatic interactions with the inhibitors. Notably, Arg-155 and Leu-159 contributed the largest stabilization energies (–246.59 kcal/mol and –78.63 kcal/mol, respectively), indicating their importance in anchoring the ligands through hydrogen bonding and hydrophobic contacts.

### 3.10. Quantum chemical descriptors

**Fig 11:**
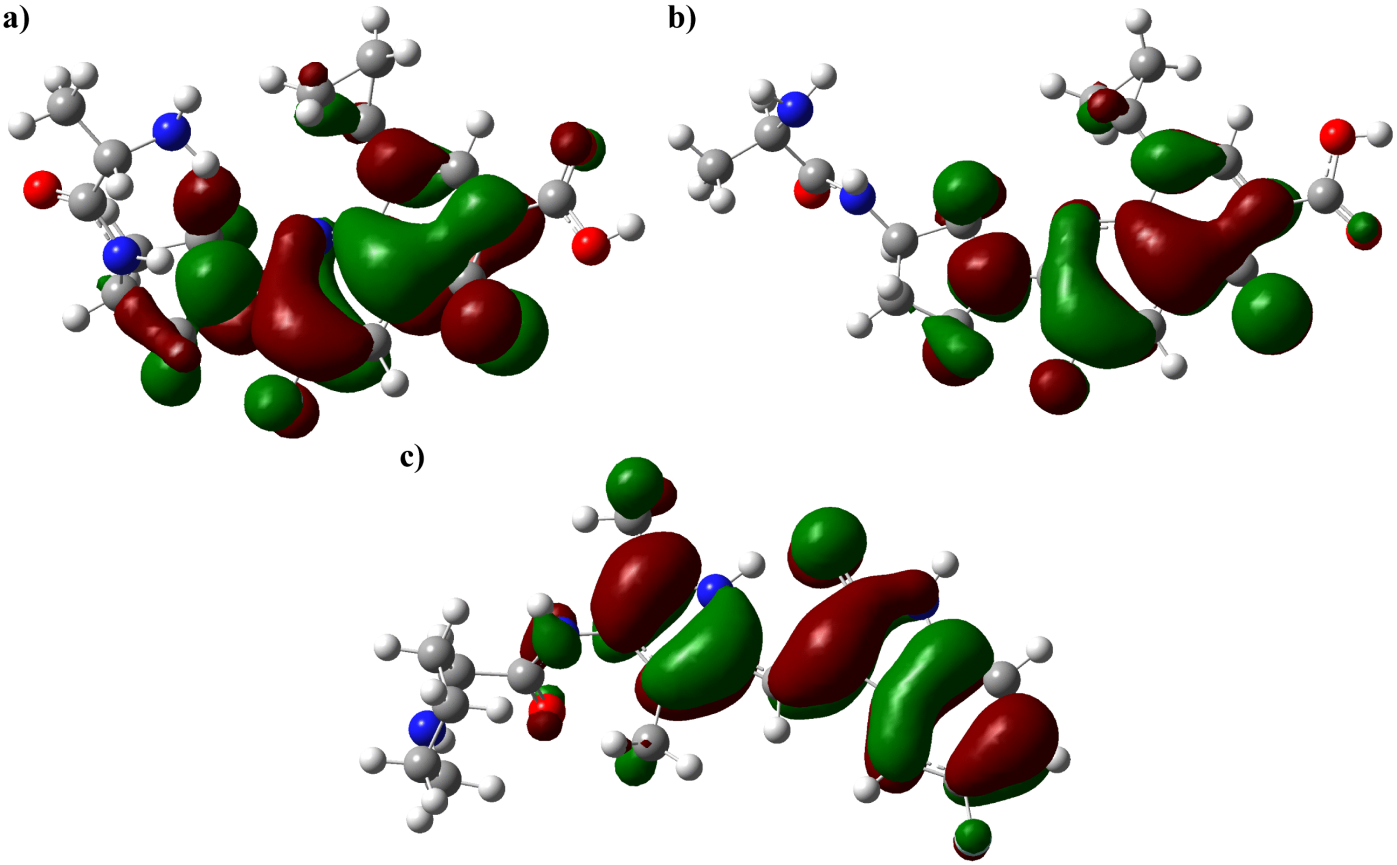
Frontier molecular orbital distributions of the selected compounds obtained from DFT calculations at the B3LYP/6-311G (d, p) level of theory. (a) CHEMBL50064, (b) CHEMBL49087, and (c) CHEMBL4117527.

Density Functional Theory (DFT) calculations were employed to investigate the frontier molecular orbitals and global reactivity descriptors of the three selected compounds (CHEMBL50064, CHEMBL49087, and CHEMBL4117527). The spatial distributions of the HOMO and LUMO orbitals are illustrated in Fig 11, while the calculated quantum chemical parameters are summarized in Table 5. The analysis revealed that CHEMBL50064 exhibited a HOMO energy of –5.93 eV and a LUMO energy of –1.67 eV, corresponding to a HOMO–LUMO energy gap of 4.26 eV. CHEMBL49087 displayed a nearly identical electronic profile (ΔE = 4.24 eV), whereas CHEMBL4117527 showed a narrower gap of 3.38 eV, indicating comparatively higher electronic reactivity. A smaller energy gap typically suggests greater chemical reactivity and enhanced ability to participate in charge transfer processes. Further, CHEMBL50064 demonstrated the highest ionization potential (5.93 eV) and chemical hardness (5.09 eV), suggesting strong resistance to electron removal and relatively stable electronic configuration. In contrast, CHEMBL4117527 possessed the lowest hardness (4.31 eV) and the highest chemical softness (0.23 eV), indicating a greater tendency to adapt its electron cloud during interactions with target residues. The electronegativity values followed the order CHEMBL50064 > CHEMBL49087 > CHEMBL4117527, implying that CHEMBL50064 has the strongest electron-withdrawing tendency, which may enhance its binding complementarity within a polar or charged binding pocket. Overall, the DFT-derived descriptors indicate that CHEMBL4117527 is the most electronically reactive among the studied compounds, potentially favouring dynamic interactions within the protein active site, whereas CHEMBL50064 and CHEMBL49087 exhibit higher chemical stability and moderate electrophilicity. These findings suggest complementary electronic characteristics that may underlie their differential binding affinities and biological activities.

**Table 5:** Calculated quantum chemical descriptors of the selected compounds using the B3LYP/6-311G (d, p) method.

	CHEMBL50064	CHEMBL49087	CHEMBL4117527
E_HOMO_	−5.93 eV	−5.91 eV	−5.24 eV
E_LUMO_	−1.67 eV	−1.67 eV	−1.86 eV
Ionization Potential	5.93 eV	5.91 eV	5.24 eV
Electron Affinity	1.67 eV	−1.67 eV	1.86 eV
Electronegativity	6.76 eV	6.75 eV	6.17 eV
Electrophilicity Index	−6.76 eV	−6.75 eV	−6.17 eV
Chemical Hardness	5.09 eV	5.08 eV	4.31 eV
Chemical Softness	0.19 eV	0.19 eV	0.23 eV

### 3.11. Toxicity predictions

Toxicity predictions from the ProTox-II analysis revealed that all three compounds exhibited low acute toxicity, with LD₅₀ values ranging from 1300 to 2000 mg/kg, placing them within toxicity class IV. Both CHEMBL50064 and CHEMBL49087 displayed LD₅₀ values of 2000 mg/kg, while CHEMBL4117527 showed a slightly lower value (1300 mg/kg), suggesting moderate oral tolerance (Table 6). None of the compounds demonstrated cytotoxic, carcinogenic, or immunotoxic behaviour, as all were predicted to be inactive across these endpoints. This indicates a favourable *in silico* safety profile for the designed compounds. Collectively, the ProTox-II predictions support the non-toxic and drug-like nature of the candidate molecules, aligning well with the previously obtained ADMET and DFT findings. The absence of major toxicity alerts further reinforces their potential as safe and stable therapeutic leads for downstream optimization and biological evaluation.

**Table 6:** Predicted toxicity parameters of the selected compounds obtained from the ProTox-II server.

	CHEMBL50064	CHEMBL49087	CHEMBL4117527
LD50	2000 mg/kg	2000 mg/kg	1300 mg/kg
Cytotoxicity	Inactive	Inactive	Inactive
Carcinogenicity	Inactive	Inactive	Inactive
Immunotoxicity	Inactive	Inactive	Inactive

## 4. Discussion

Janus kinase 3 (JAK3) plays a distinctive role among the JAK family members due to its selective expression in hematopoietic cells and involvement in γc cytokine receptor–mediated signalling, which governs lymphocyte development, proliferation, and immune regulation. Aberrant JAK3 activation has been reported in several hematologic malignancies and solid tumors, highlighting its relevance as a potential therapeutic target [1,5,36]. While pan-JAK inhibitors such as tofacitinib and ruxolitinib have demonstrated clinical success in autoimmune diseases, selective inhibition of JAK3 remains limited because of the high sequence similarity within ATP-binding pockets of JAK1, JAK2, and JAK3 [6,7].

The present study introduces an integrated computational framework combining machine learning (ML) with molecular docking, molecular dynamics (MD) simulations, and free-energy calculations to identify and validate potential JAK3 inhibitors. Compared with earlier computational studies that relied primarily on predictive modelling [15,16], this work incorporates multi-tier validation using long-timescale MD simulations and energetic decomposition.

The Random Forest model developed here showed strong predictive capability (AUC = 0.80; F1 = 0.92), consistent with accuracies reported for kinase-target ML models such as JAK2 and BTK [37]. Virtual screening of the ChEMBL library identified 400 high-confidence candidates, of which CHEMBL4117527, CHEMBL49087, and CHEMBL50064 exhibited the most favourable docking affinities (≤ –9.0 kcal mol ⁻ ¹). The observed binding of these ligands to key residues Leu108, Arg156, and Asp170 matches those reported for known JAK3 inhibitors PF-06651600 and RB1 [8,13].

ADMET and ProTox-II analyses indicated favourable pharmacokinetic and safety profiles, consistent with current efforts to design safer, more selective JAK3 inhibitors [14,38]. MD simulations confirmed the dynamic stability of the protein–ligand complexes over 200 ns trajectories; all maintained low RMSD values (0.10–0.20 nm) and stable hydrogen-bond occupancy [39,40].

Binding-free-energy calculations further supported CHEMBL4117527 as the most stable complex, with ΔGbind values of –29.5 kcal mol ⁻ ¹ (MM/GBSA) and –26.1 kcal mol ⁻ ¹ (MM/PBSA), consistent with typical high-affinity kinase inhibitors [17,41]. Residue decomposition identified Leu31, Val39, Arg155, and Leu159 as major contributors to stability, in agreement with earlier reports highlighting hydrophobic pocket residues as determinants of JAK3 selectivity [42].

Quantum-chemical analysis using density-functional theory (DFT) provided complementary insights into electronic reactivity. CHEMBL4117527 displayed the smallest HOMO–LUMO energy gap (3.38 eV) and highest chemical softness (0.23 eV), implying a greater propensity for charge transfer within the active site—an observation consistent with prior DFT-guided inhibitor design studies [21,43].

Compared with existing work, this study contributes three main advances: (i) integration of ML-based screening with physics-based post-validation in a single pipeline for JAK3; (ii) use of dual free-energy approaches (MM/GBSA and MM/PBSA) for cross-validation; and (iii) linkage of these energetic findings with orbital-level DFT descriptors.

Nonetheless, certain limitations should be acknowledged. The use of 2D fingerprints in ML modelling may overlook 3D conformational and stereo electronic features that influence kinase selectivity. MM/GBSA and MM/PBSA methods approximate solvent and entropic effects and therefore may underestimate total binding energies. Finally, the absence of experimental validation is a key limitation; biochemical assays and cell-based evaluations are required to verify inhibitory potency and cytotoxic selectivity.

Future work should include in vitro and in vivo validation of the identified compounds to confirm their biological activity. Free-energy perturbation (FEP) and meta dynamics simulations could refine energy predictions, while analog development of CHEMBL4117527 through structure–activity relationship (SAR) exploration may yield next-generation JAK3 inhibitors with improved efficacy and safety. Expanding this computational workflow to other JAK isoforms could further clarify selectivity determinants across the kinase family.

## 5. Conclusion

This study establishes an integrated machine learning–guided and simulation-validated framework for the discovery of selective JAK3 inhibitors. By combining data-driven virtual screening with molecular docking, long-timescale molecular dynamics simulations, and free energy and quantum chemical analyses, the research provides a multi-level validation strategy that bridges predictive modeling with biophysical accuracy. The findings identify CHEMBL4117527 as a promising JAK3 inhibitor exhibiting favorable stability, strong binding energetics, and optimal pharmacokinetic and safety profiles. These results advance current knowledge by demonstrating that AI-assisted modeling, when coupled with physics-based methods, can accelerate kinase-focused drug discovery with improved reliability. Looking forward, this approach can be extended to experimental validation, structure–activity optimization, and clinical translation, offering a scalable pathway for developing next-generation therapeutics targeting JAK3-driven cancers and immune disorders.

## Supporting information

S1 Data **Figure S1.** The alignment of trajectory snapshots to analyze protein-ligand stability. a) Co-crystal structure, b) CHEMBL50064, c) CHEMBL49087, and d) CHEMBL4117527. **Figure S2.** The molecular interactions of hit compounds before and after simulation. Panel A shows the interactions before simulation, and Panel B shows the interactions after simulation.
(DOCX)
