# Supplementary material for: Machine learning-guided identification and simulation-based validation of potent JAK3 inhibitors for cancer therapy
Source: PLoS One. 2025 Dec 12;20(12):e0338777. doi: 10.1371/journal.pone.0338777 (PMC12700429; doi:10.1371/journal.pone.0338777)
Supplement: S1 Data — Figure S1. The alignment of trajectory snapshots to analyze protein-ligand stability. a) Co-crystal structure, b) CHEMBL50064, c) CHEMBL49087, and d) CHEMBL4117527. Figure S2. The molecular interactions of hit compounds before and after simulation. Panel A shows the interactions before simulation, and Panel B shows the interactions after simulation. (DOCX) [file pone.0338777.s001.docx]

| 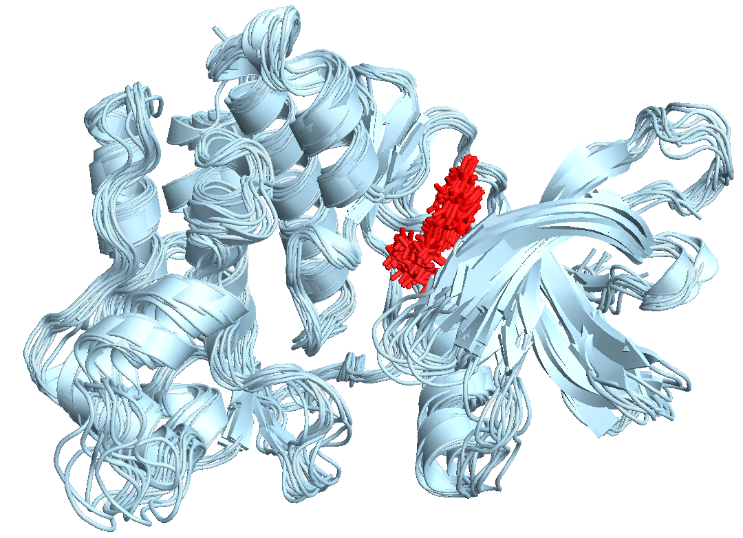  **a)** | 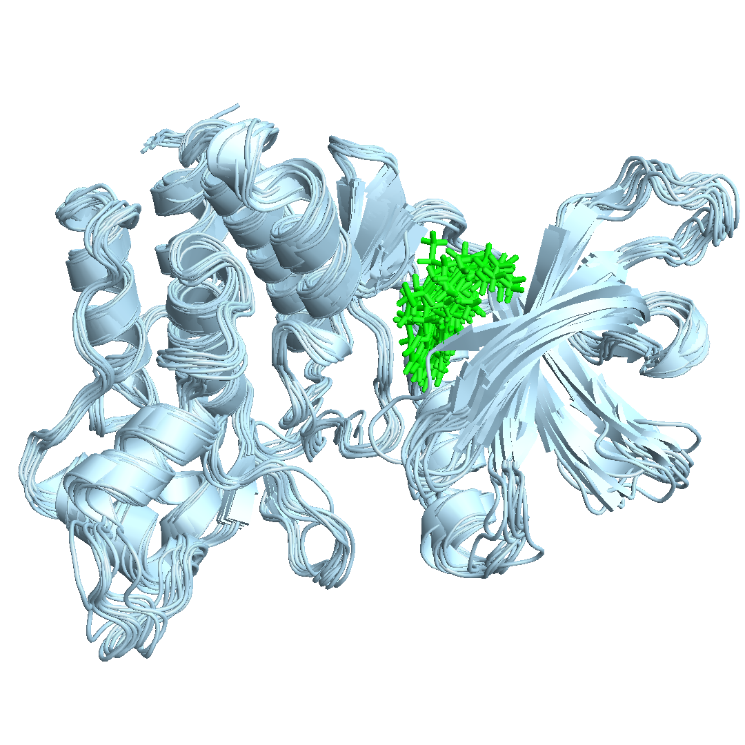  **b)** |
| --- | --- |
| 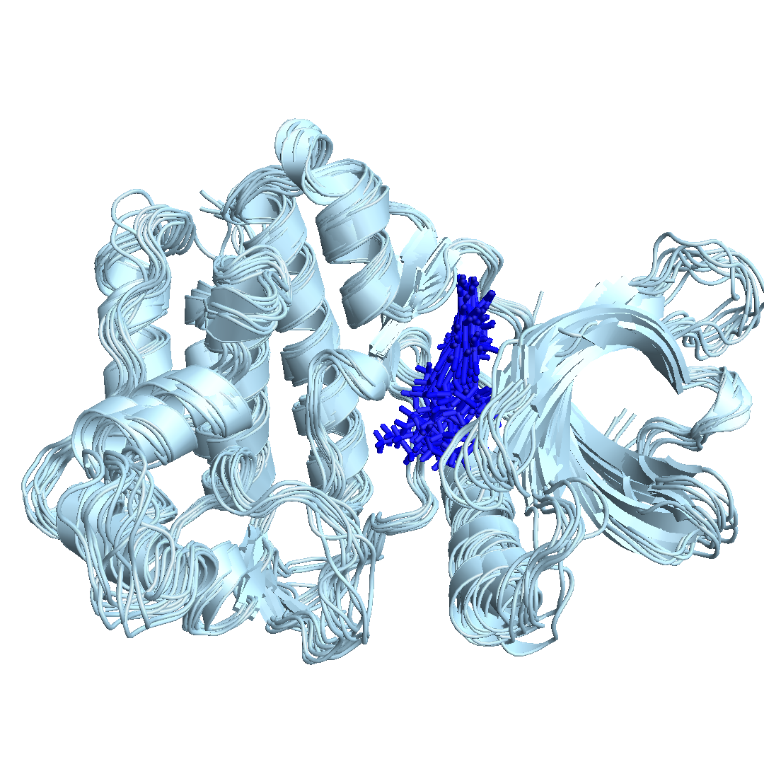  **c)** | 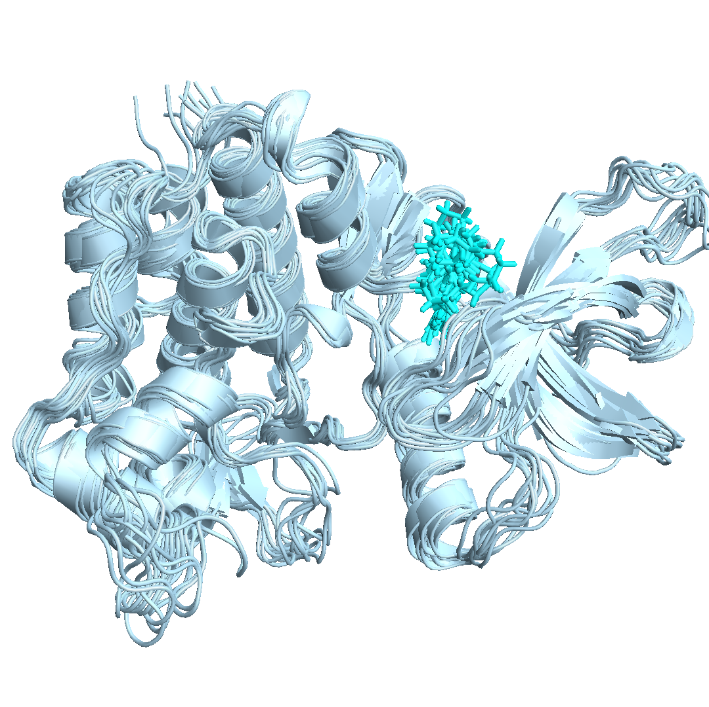  **d)** |

Figure S1. The alignment of trajectory snapshots to analyze protein-ligand stability. a) Co-crystal structure, b) CHEMBL50064, c) CHEMBL49087, and d) CHEMBL4117527.

| CHEMBL50064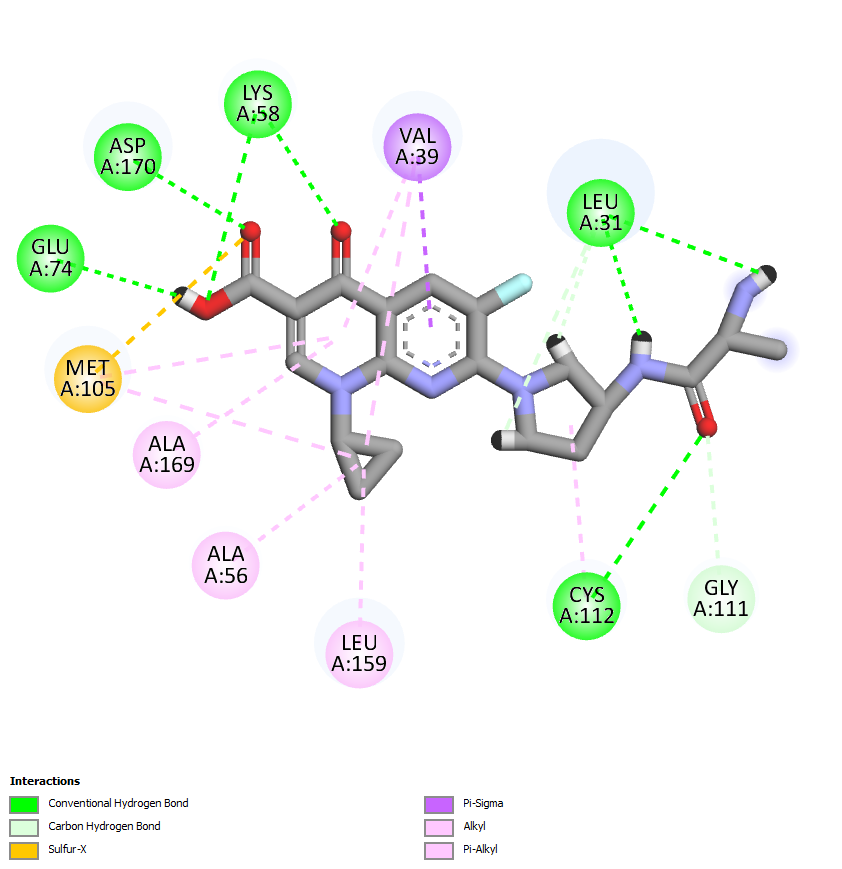  **A)** | 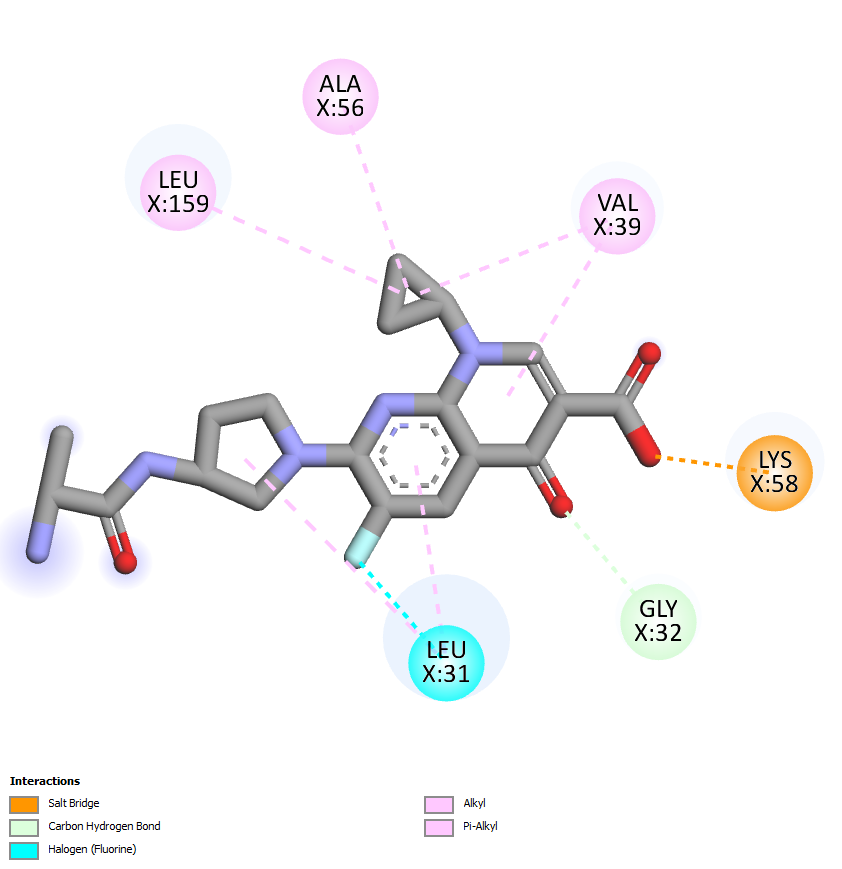  **B)** |
| --- | --- |
| 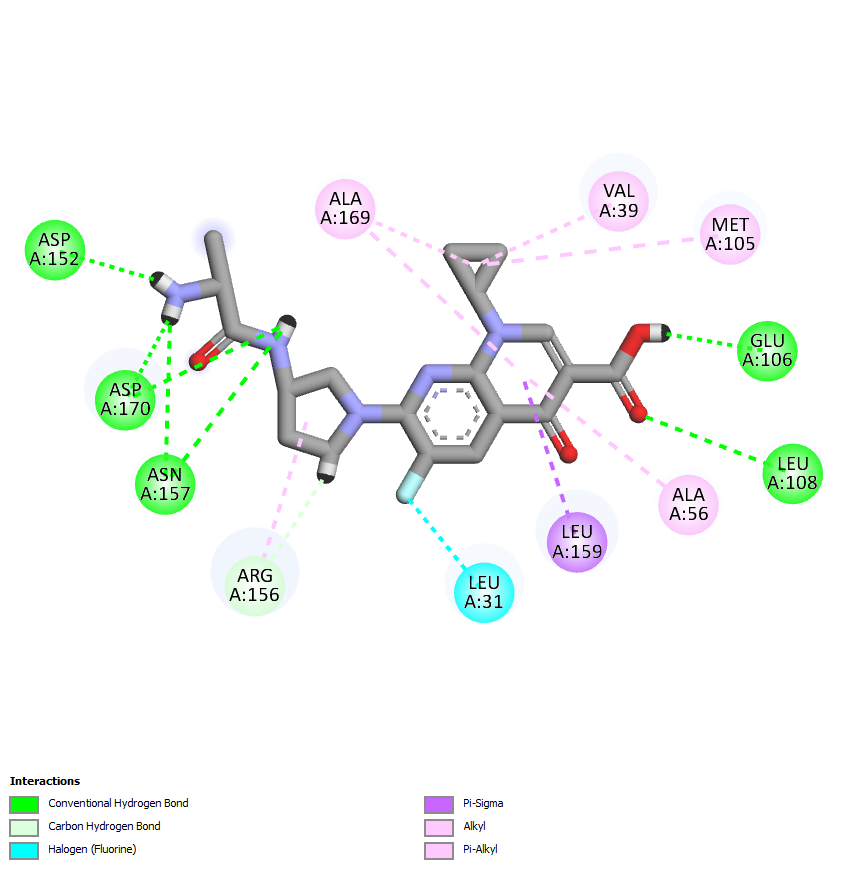  CHEMBL49087 | 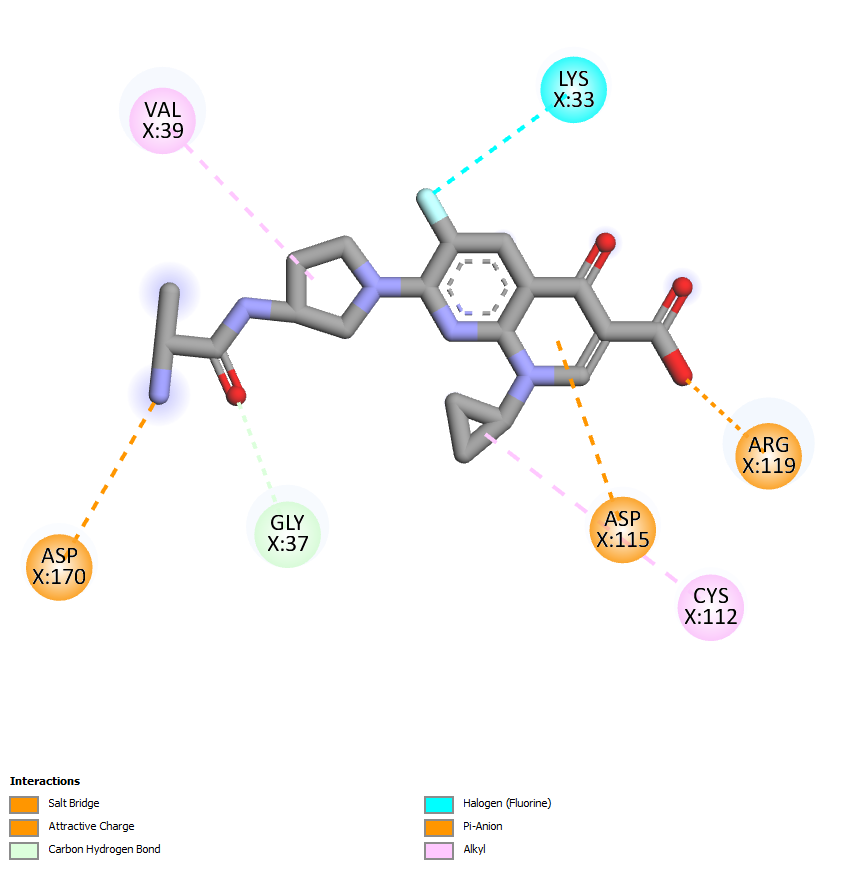 |
| CHEMBL4117527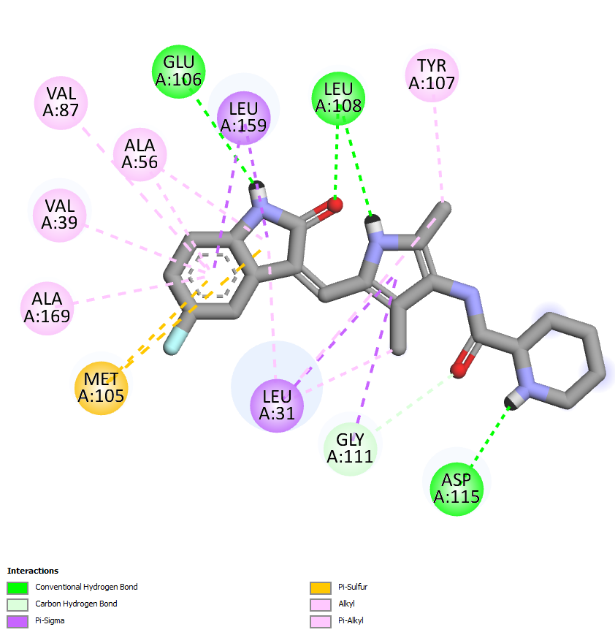 | 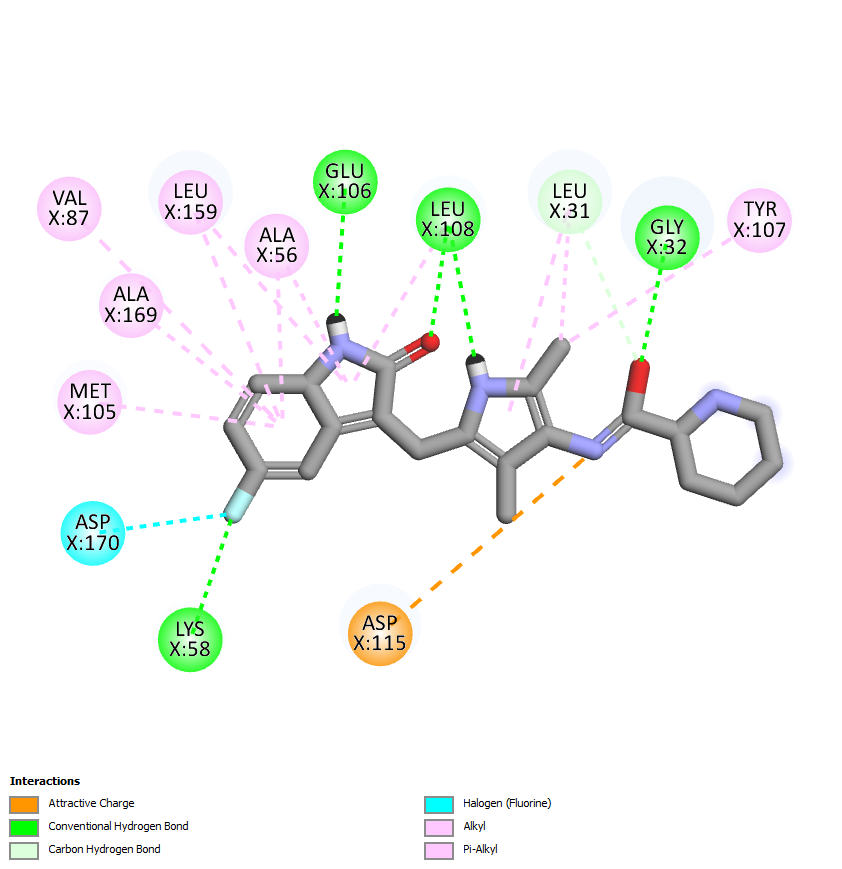 |

Figure S2. The molecular interactions of hit compounds before and after simulation. Panel A shows the interactions before simulation, and Panel B shows the interactions after simulation.
